# Design of a study on suboptimal cognitive acts in the diagnostic process, the effect on patient outcomes and the influence of workload, fatigue and experience of physician

**DOI:** 10.1186/1472-6963-9-65

**Published:** 2009-04-21

**Authors:** Laura Zwaan, Abel Thijs, Cordula Wagner, Gerrit van der Wal, Daniëlle RM Timmermans

**Affiliations:** 1 EMGO Institute for Health and Care Research, Department of Public and Occupational Health, VU University Medical Center, Amsterdam, The Netherlands; 2 Department of Internal Medicine, VU University Medical Center, Amsterdam, The Netherlands; 3 NIVEL, Netherlands Institute for Health Services Research, Utrecht, The Netherlands

## Abstract

**Background:**

Diagnostic error is an important error type since diagnostic adverse events are regularly judged as being preventable and the consequences are considered to be severe. Existing research often focuses on either diagnostic adverse events or on the errors in diagnostic reasoning. Whether and when an incorrect diagnostic process results in adverse outcomes has not been studied extensively. The present paper describes the design of a study that aims to study the relationship between a suboptimal diagnostic process and patient outcomes. In addition, the role of personal and circumstantial factors on the quality of the diagnostic process will be examined.

**Methods/Design:**

The research questions were addressed using several data sources. First, the differential diagnosis was assessed concurrently to the diagnostic process. Second, the patient records of 248 patients suffering from shortness of breath were reviewed by expert internists in order to reveal suboptimal cognitive acts and (potential) consequences for the patient. The suboptimal cognitive acts were discussed with the treating physicians and classified with the taxonomy of unsafe acts. Third, workload, fatigue and work experience were measured during the physicians work. Workload and fatigue were measured during the physicians shift using the NASA tlx questionnaire on a handheld computer. Physicians participating in the study also answered questions about their work experience.

**Discussion:**

The design used in this study provides insight into the relationship between suboptimal cognitive acts in the diagnostic process and possible consequences for the patient. Suboptimal cognitive acts in the diagnostic process and its causes can be revealed. Additional measurements of workload, fatigue and experience allow examining the influence of these factors on the diagnostic process. In conclusion, the present design provides a method with which insights in weaknesses of the diagnostic process and the effect on patient outcomes can be studied and opportunities for improvement can be obtained.

## Background

In order to deliver good quality of care a correct diagnosis based on complaints and symptoms of a patient is required. Physicians arrive at a diagnosis based on the patients' history, a physical examination and additional information such as results of laboratory tests and imaging techniques. From a large amount of information the physician has to select the information relevant for diagnosing the patient correctly. Based on this information different diagnoses are considered, investigated and subsequently confirmed or ruled out. Sometimes, this is a relatively simple task while in other cases it is complicated and a lot of decision making skills are required. International studies demonstrate that a substantial percentage of the diagnoses are incorrect [1,2]. The percentages found in different studies vary widely [2,3]. Berner & Graber (2008) [4] show that the rate of diagnostic error depends on medical specialty. They summarize that in perceptual specialties (e.g. pathology and radiology) diagnostic errors appear in 2–5%, while in other specialties it occurs in 10–15% of the cases. In several post-mortem studies the percentage increases to 40% [5].

An incorrect diagnosis can have major effects on treatment and therefore on the outcome of treatment [6] and can even lead to a patient's death. The mortality caused by diagnostic failure is higher than for any other type of medical error [7,8]. Patients are also aware of diagnostic errors as research shows that 30% or more of the malpractice claims are about diagnostic adverse events [9-12]. It is also known that at the emergency department, out of all medical errors, patients are most concerned about being misdiagnosed [13]. For both patients and physicians diagnostic error is important. However, studying diagnostic error is difficult.

Existing research on diagnostic error can be divided into two main research areas. The first area, post mortem studies and retrospective adverse event studies [2,3,14,15], focuses on the adverse outcomes and count and describe incorrect diagnoses and study the adverse outcomes for the patient. Another field of research focuses on cognitive error in the diagnostic process [16,17]. According to the model of unsafe acts, cognitive errors can occur due to intended or unintended actions [18]. Unintended actions are slips and lapses and are described as errors which result from a failure in the execution and/or storage of an action sequence, regardless of whether or not the plan which guided them was adequate to achieve its objects [18]. An example of a slip is applying for an incorrect test due to checking the wrong box on the laboratory form. The intended actions are mistakes and violations. Mistakes occur when a plan to perform an action is inadequate to achieve its intended outcome [18], for example when a physician applies an incorrect laboratory test while he thought it was the correct test to rule out a certain disease. Violations are defined as any behavior that deviates from accepted procedures, standards and rules such as not applying a test while knowing it would be better to apply for the test. Since slips and lapses occur in the execution phase of a task, they are more influenced by high workload and fatigue [19-21] resulting for example in distraction from a routine event. Since mistakes are errors in knowledge and interpretation, they are likely more influenced by work experience [18]. Most cognitive errors do not lead to adverse outcomes. However, the relationship between cognitive error in diagnostic reasoning, circumstantial factors (workload, fatigue and work experience) and the occurrence of adverse events has not yet been studied.

The present study has several goals which will be studied in a specific patient group suffering from shortness of breath (dyspnea). The aims are:

1. To reveal suboptimal cognitive acts in the diagnostic process and their causes and to determine the relationship between suboptimal cognitive acts and patient outcomes.

2. To establish the relationship between workload, fatigue and work experience and suboptimal cognitive acts.

## Methods/Design

### Patient group

Patients arriving at the hospital suffering from shortness of breath (dyspnea) who were seen by a physician (mostly residents) of the participating specialties were included in the study. As the study focuses on suboptimal cognitive acts a specific patient group was selected since it is easier to discover deviations from the optimal process when the group is homogenous and the optimal diagnostic process can be defined more clearly. As this study focuses on the diagnostic process and patients were included prospectively it was crucial to select patients based on a symptom rather than a disease. A large variety of diseases can cause shortness of breath and the levels of difficulty of establishing a correct diagnosis vary.

### Hospital selection

The study took place in five hospitals in the Netherlands in the departments of internal medicine, cardiology and pulmonology between May 2007 and May 2008. A random sample of hospitals located within a reasonable traveling distance (2 hours) of the researcher was invited to participate in the study. All participating hospitals were acute care hospitals, one university hospital, two tertiary medical teaching hospitals and two general hospitals. The organization of the departments varied between the hospitals e.g. in some hospitals residents from the pulmonology and cardiology were not exchangeable with the residents of the internal medicine department while in other hospitals all residents worked alternating shifts in the departments of internal medicine, pulmonology as well as cardiology.

#### 1. Determining suboptimal cognitive acts in diagnostic process and patient outcomes

**Figure 1:**
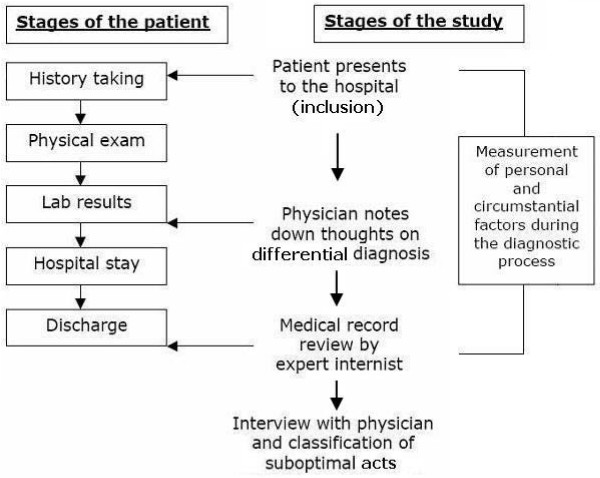
**An overview of the data gathering process**.

The diagnostic process was assessed by different data sources which together added up to a more complete assessment of the diagnostic process. First, information of the differential diagnoses was gathered from the treating physician concurrently to diagnosing the patient. The second data source was a retrospective record review of the diagnostic process and patient outcomes. The questionnaire used in the record review was based on an optimal diagnostic process for dyspnea patients developed using a Delphi method. The record review revealed suboptimal cognitive acts, which are cognitive errors using a very liberal threshold and therefore includes a lot of minor deviations from the optimal process. The record review was followed by a short meeting with the treating physician to elucidate the suboptimal cognitive acts as assessed by the expert reviewers to have occurred during the diagnostic process. The data sources are described in detail below and see Figure 1 for an overview of the data gathering process.

##### Information about differential diagnosis

**Figure 2:**
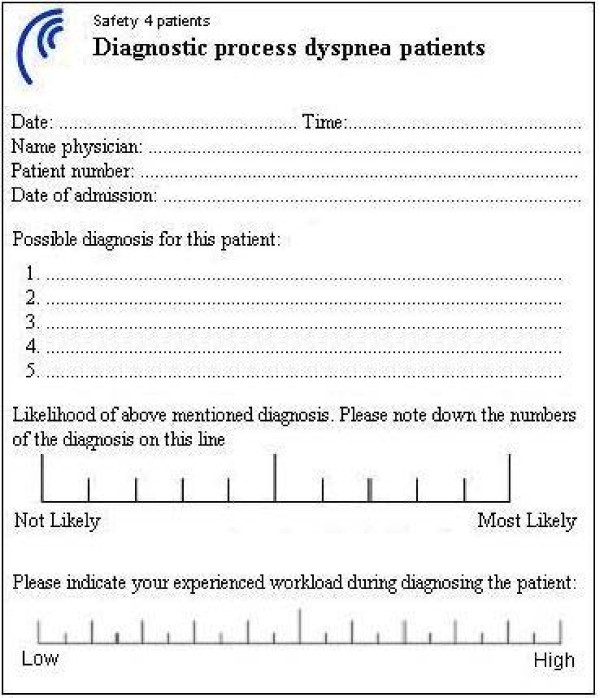
**Form with questions about differential diagnosis and workload**.

The treating physicians were asked to note down their differential diagnoses in order of probability. After the treating physician decided to admit the patient to the hospital they were asked to fill out a small questionnaire (see Figure 2). They noted down what possible underlying diagnoses could cause the dyspnea in the patient, subsequently they indicated how likely they thought these diagnoses were.

##### Record review of diagnostic process

###### Optimal diagnostic process

Seven expert internists participated in developing the optimal diagnostic process for dyspnea patients in a two stage Delphi method. First, all participating expert internists received a questionnaire with twelve cases of patients who were admitted to the hospital suffering from dyspnea. The questions focused on the important decisions during the diagnostic process. After analyzing the questionnaires a first draft of the optimal process was developed by a researcher (LZ) and an internist (AT). During a second meeting with the expert internists, the draft of the optimal diagnostic process was discussed and commented on by the expert internists and a researcher (DT). The comments of the internists and researcher were processed and the final version of the optimal diagnostic process was completed and agreed upon by the expert internists.

###### Review questionnaire

The questionnaire used to review the patient records was based on the optimal diagnostic process. The diagnostic process was assessed according to the stages of the diagnostic process; (1) History taking, (2) physical examination, (3) laboratory results, (4) imaging techniques, (5) outlining a diagnosis, (6) starting the treatment and (7) verification of diagnosis and treatment during the patients' stay. The first four stages are information gathering stages, and the last three are stages of information integration. The information gathering stages were reviewed on two criteria (1) whether the correct information was gathered and (2) whether the information was interpreted correctly. The information interpreting stages were reviewed on interpretation only.

####### Information gathering stages

**Figure 3:**
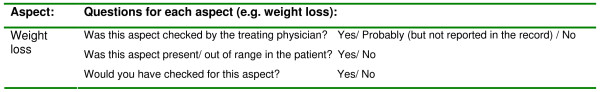
**Questions asked about all aspects in history taking, physical examination, laboratory and imaging techniques**.

For the information gathering stages (history taking, physical examination, laboratory results and imaging techniques) all relevant aspects for patients with dyspnea were reviewed (i.e. weight loss, medication use, allergies, temperature, listening to the lungs, CT scan). For each aspect the expert reviewers checked the patient record and answered the questions in Figure 3.

**Figure 4:**
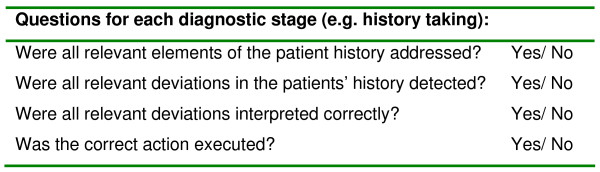
**Questions the record reviewers were asked after each of the 7 diagnostic stages**.

After every information gathering stage several questions about the interpretation of the gathered information were asked which included the questions in Figure 4.

####### Information integration stages

The review questionnaire assessed the information integration stages systematically by questions about the decisions of the physician and the decisions made on the diagnosis and treatment. Examples of the questions were 'Did the treating physician verify the outcomes of the treatment on time?' and 'Did the treating physician integrate all information into a correct diagnosis?'. Furthermore, some questions to obtain additional information were asked in order to obtain more information about the circumstances under which the suboptimal cognitive acts occur, such as: patient factors, supervision and the number of physicians involved in the diagnostic process.

###### Reviewer selection and reviewing process

Four of the expert internists were invited to participate as reviewers in the reviewing process of the patient records. These expert internists were recruited from a group of fifty-five physicians who participated as reviewers in a large retrospective study on the occurrence of adverse events in the Netherlands[22]. The selection criteria were:

• At least 10 years of post-graduate work experience in internal medicine

• A good reputation amongst colleagues

• Retired for no longer than 5 years at time of selection as a reviewer

• Interest in and experience with patient safety research

The reviewers attended a one-day training program which was led by a researcher (LZ) and an internist (AT). The topics addressed were the study protocol (e.g. background and research goals), the review questionnaire which was used to review the diagnostic process and the computer program in which the review questionnaire was programmed. Subsequently, two half-day practice meetings were organized during which the expert internists practiced with the review questionnaire and the computer system. In addition, after reviewing 10–15 records in the hospitals, another training session was organized to discuss questions and difficulties the reviewers experienced. The expert internist reviewers were paid a small fee for their work hours and compensated for their expenses.

The record review procedure took place in the hospitals. The review questionnaire was transformed into an electronic template with the program Blaise [23]. The expert reviewers entered the data directly into the program which was installed on laptop computers. A researcher was always present during the reviewing process to answer questions about the study protocol and for technical support.

###### Information from the treating physician and classification of suboptimal cognitive acts

Two days to eight months after the patients' discharge the patient records were reviewed, a researcher (LZ) discussed the suboptimal cognitive diagnostic acts found in the record review with the physicians who treated the patients. In a few cases the meeting was eight months after the patients' discharge due to the time consuming process of obtaining and reviewing the records and subsequently planning a meeting with the treating physician. During this meeting with the treating physicians, the physicians clarified their decisions and provided additional information on the assessed suboptimal cognitive acts. The researcher (LZ) was instructed by the expert reviewers about the questions that needed to be asked. Based on the findings of the record review and the interview with the treating physician the suboptimal cognitive acts were classified by a researcher (LZ) and an internist (AT) into the error categories: slip, lapse, mistake and violation of the model of unsafe acts [18].

###### Suboptimal cognitive acts

The main outcome measurements were the suboptimal cognitive acts found in the record review. An act was defined as suboptimal if (1) it was indicated as such by the internist reviewers in the record review and (2) it was not contradicted during the meeting with the physician. Suboptimal cognitive acts were for instance: not ordering a specific test or ordering an unnecessary test, misinterpreting test results etc. Subsequently, after defining the suboptimal cognitive acts a researcher (LZ) and an internist (AT) described all suboptimal cognitive acts. For all suboptimal acts it was determined in which diagnostic stage of the diagnostic process they occurred and what the cause of the act was using the taxonomy of unsafe acts[18].

#### 2. Measurement of workload, fatigue and work experience

The second source of data was measurements of workload, fatigue and work experience of the treating physician, measured during the patients hospital stay.

##### Workload

Workload was assessed in two ways. First, at the patients' admission the physician answered a question (scale 1 to 20) about the level of the subjective workload (see Figure 2).

Second, the workload of the physicians during the course of the day was measured. In each hospital every two hours physicians filled out the level of their subjective workload using the NASA tlx [24] questionnaire (short version) on a handheld computer. The physicians were notified by an alarm on the handheld computer when to fill out the questionnaire. The NASA tlx exists of 6 subjective dimensions: mental demand, physical demand, temporal demand, performance, effort and frustration (all with scale 1–20).

##### Fatigue

When the physicians filled out the NASA tlx workload questionnaire on the handheld computer, they also answered one question about their level of fatigue (scale 1–20) to measure the fluctuation of the level of fatigue during the working day.

##### Work experience

All physicians who included patients in the study or were involved in the care for the patients were asked about their work experience by 4 questions.

How long have you worked:

1. as a physician after you obtained your medical degree?

2. in the hospital you are currently employed?

3. in the hospital department you are currently employed?

4. in your current specialization (internal medicine, cardiology or lung diseases)?

### Sample size

As various research questions are studied and data analysis is mainly descriptive we based our sample size on the distribution of causes of the suboptimal cognitive acts. In order to obtain a substantial number of suboptimal cognitive acts in each category (slip, lapse, mistake, violation and patient record problem) a confidence interval was calculated assuming an equal distribution of suboptimal cognitive acts in each category (20% of the suboptimal cognitive acts in each category). Based on a pilot review of several patient records by an internist (AT), it was expected to find an average of 1,5 suboptimal acts per patient record which would mean that 250 patient records (= 375/1,5) are required in the study.

Using a binomial distribution and 375 suboptimal acts, the confidence interval is as follows:

*CI *= 0,20 ± 0,040 ≈ 0,16 - 0,24. This is a respectable confidence interval.

### Confidentiality and ethical approval

The review board of the VU medical center approved the research protocol. All participating hospitals granted approval to participate. The reviewers and researchers involved in the data collection signed a confidentiality agreement to maintain the secrecy of the data. Furthermore, the expert reviewers did not review records in hospitals where they had been employed in the course of their career. All patients who were included in the study gave informed consent to review their patient record. All patients were assigned to a unique study number in order to keep the patients' identity confidential. Patient identifiers were kept in a dataset separately from the primary database. Patients' names were not included in the database and after completion of the data collection and analysis, medical record identifiers were destroyed.

### Reliability study

To assess the reliability of the review process, a random sample of 5% of the records were independently reviewed by two expert reviewers.

## Data Analysis

### Statistical analysis

During and after the data collection data-checks were executed to identify and correct out-of-range answers and inconsistent responses. After the data collection was completed the data were extracted from the Blaise program and imported into SPSS 15.0 for windows.

To answer the main questions descriptive statistics such as cross tables and frequencies were used. Subsequently, specific research questions will be answered with more specific analyses.

## Discussion

The present paper describes a study on the diagnostic process and suboptimal cognitive acts in patients presenting to a hospital with dyspnea. The method is particularly innovative as data were collected both retrospectively as well as concurrently with the diagnostic process of the treating physician and therefore provides a new approach on studying the diagnostic process.

### Strengths and limitations

#### Strengths

Studying the diagnostic process is difficult. First of all, the reasoning process occurs in the physicians' head and is difficult to track. Secondly, cognitive errors often still lead to a correct diagnosis while a correct diagnostic process can lead to an incorrect diagnosis (e.g. because of unusual presentation of the disease or a very rare disease). The present paper describes a method which makes it is possible to study the whole diagnostic process using both retrospective and prospective data sources. An essential aspect of this method is the involvement of the treating physicians. This is particularly interesting as in most studies conducted so far, the treating physicians were not involved. The contribution of the treating physicians provides information about the diagnostic process that could not be obtained otherwise. As the treating physicians explained why certain decisions were made, more precise information was gathered. With this information it was possible to classify the causes using the model of unsafe acts[18]. The model of unsafe acts provides a valuable classification of the causes of the suboptimal cognitive acts as it is a well-known taxonomy for human error.

Another important aspect of the study is the measurement of workload, fatigue and work experience which provides more insight into aspects that influence the diagnostic process. An advantage is that workload was measured both during the diagnostic process as well as throughout the physicians' working day. In this way the measurements of the general workload could be compared with the workload the physicians experienced during the diagnostic process and provides a baseline for the interpretation of the workload during the diagnostic process of a specific patient.

To reduce the influence of subjectivity, only highly trained expert reviewers with an extensive work experience were selected. Additionally, the subjectivity was also minimized by the meetings with the treating physicians about the suboptimal cognitive acts. They gave more information about the patient, explained why a certain decision was made and whether any other circumstances were present, for example a patient not wishing to undergo certain tests or hospital policies not allowing the physician to request certain tests at night. Also, the review questionnaire was developed very carefully using an optimal diagnostic process obtained by a two-stage Delphi method.

#### Limitations

The development of an optimal diagnostic process for patients with dyspnea is difficult as there are no objective criteria. Therefore, even though a solid strategy (Delphi method) was used we cannot consider the optimal diagnostic process as a golden standard.

The present study uses a record review as one of the data sources. This has three main limitations. Firstly, despite our efforts to make the record review as objective as possible by including a panel of experts, there is still the possibility of subjectivity as we rely on the reviewers clinical knowledge and their judgment. We did assess interrater reliability. Secondly, the retrospective review study uses medical records as a source of data. The information available in a patient record is limited and may not be sufficient to reveal all suboptimal cognitive acts. Thirdly, a limitation often mentioned in record review studies is hindsight bias. Looking back it is difficult not to get influenced by knowledge of the outcomes. Using the present method this could also be the case as the expert internist reviewers reviewed the patient record retrospectively. However, in most cases the suboptimal cognitive acts found in the study did not cause any patient harm and therefore, the expert internist reviewers could not be influenced in their judgment by the patient outcomes.

## Conclusion

In conclusion, this study provides a new approach on studying the diagnostic process. With this combination of methods the entire diagnostic process can be studied and suboptimal diagnostic acts that occurred during the diagnostic process are revealed and the causes and the consequences can be analyzed. As opposed to earlier studies, the present study involves the treating physician. This allows the causes to be studied extensively. In addition, this study provides insights into the diagnostic process, elucidating where in the process most suboptimal cognitive acts occur and which of those acts are likely to cause harm. This information could be used to develop interventions targeting specific parts of the diagnostic process. The measurements of workload, fatigue and work experience provides valuable information on the circumstances under which physicians diagnose their patients and to what extent those circumstances influence the diagnostic process.

## Competing interests

The authors declare that they have no competing interests.

## Authors' contributions

LZ drafted the manuscript, developed the study protocol and instruments. AT contributed to the design and conception of the study and critically read the manuscript. CW contributed to the design and conception of the study and critically read the manuscript. GW contributed to the design and conception of the study and critically read the manuscript. DT is general supervisor of this study and contributed to the design and conception of the study and drafted the manuscript. All authors gave their approval of the final version of the manuscript.

## Pre-publication history

The pre-publication history for this paper can be accessed here:


